# Supplementary figures and images for: Conservation of the Direct and Indirect Pathway Dichotomy in Mouse Caudal Striatum With Uneven Distribution of Dopamine Receptor D1- and D2-Expressing Neurons
Source: Front Neuroanat. 2022 Feb 4;16:809446. doi: 10.3389/fnana.2022.809446 (PMC8854186; doi:10.3389/fnana.2022.809446)

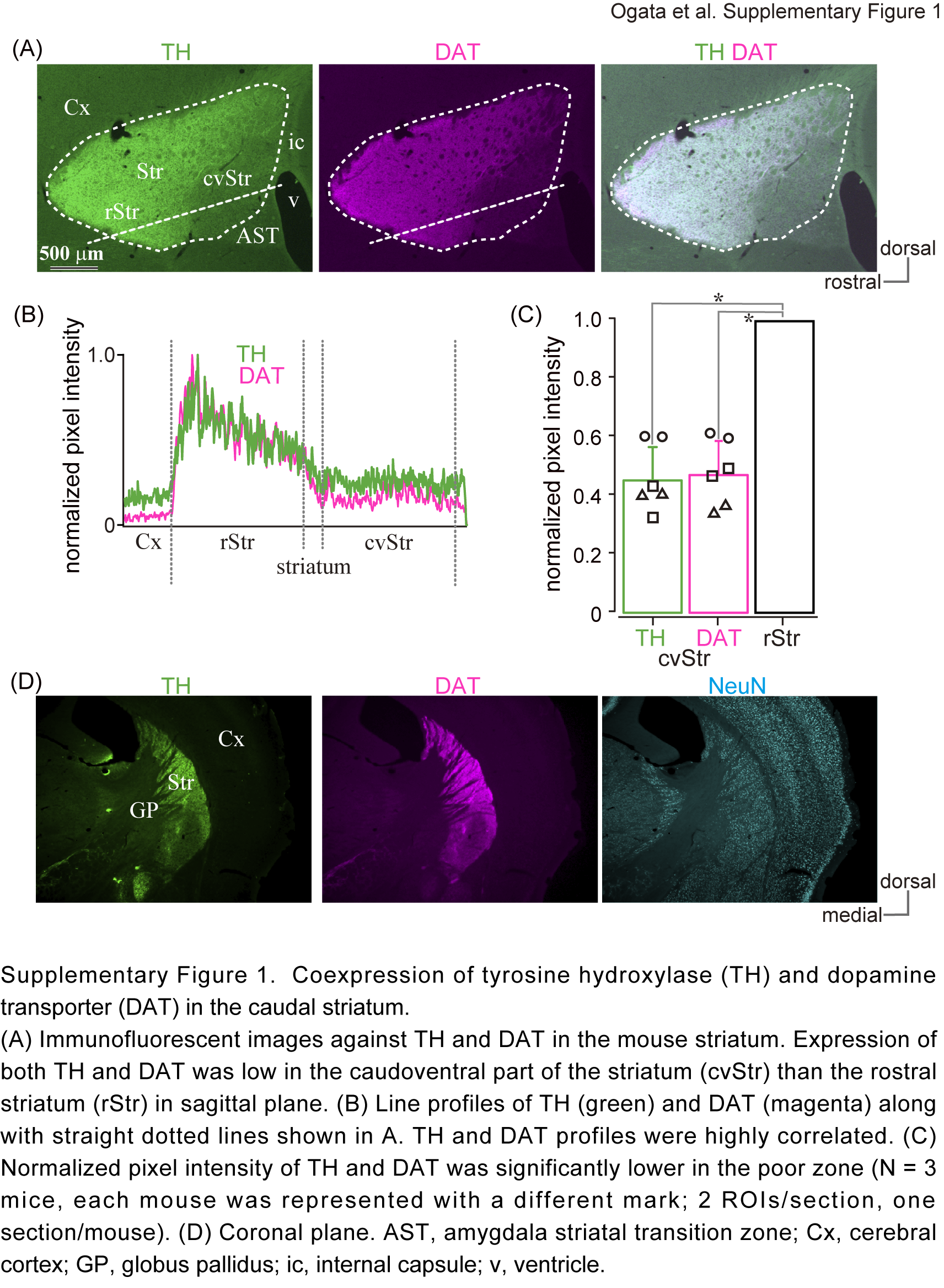

Supplement: Supplementary file 1 [file Image_1.tif]

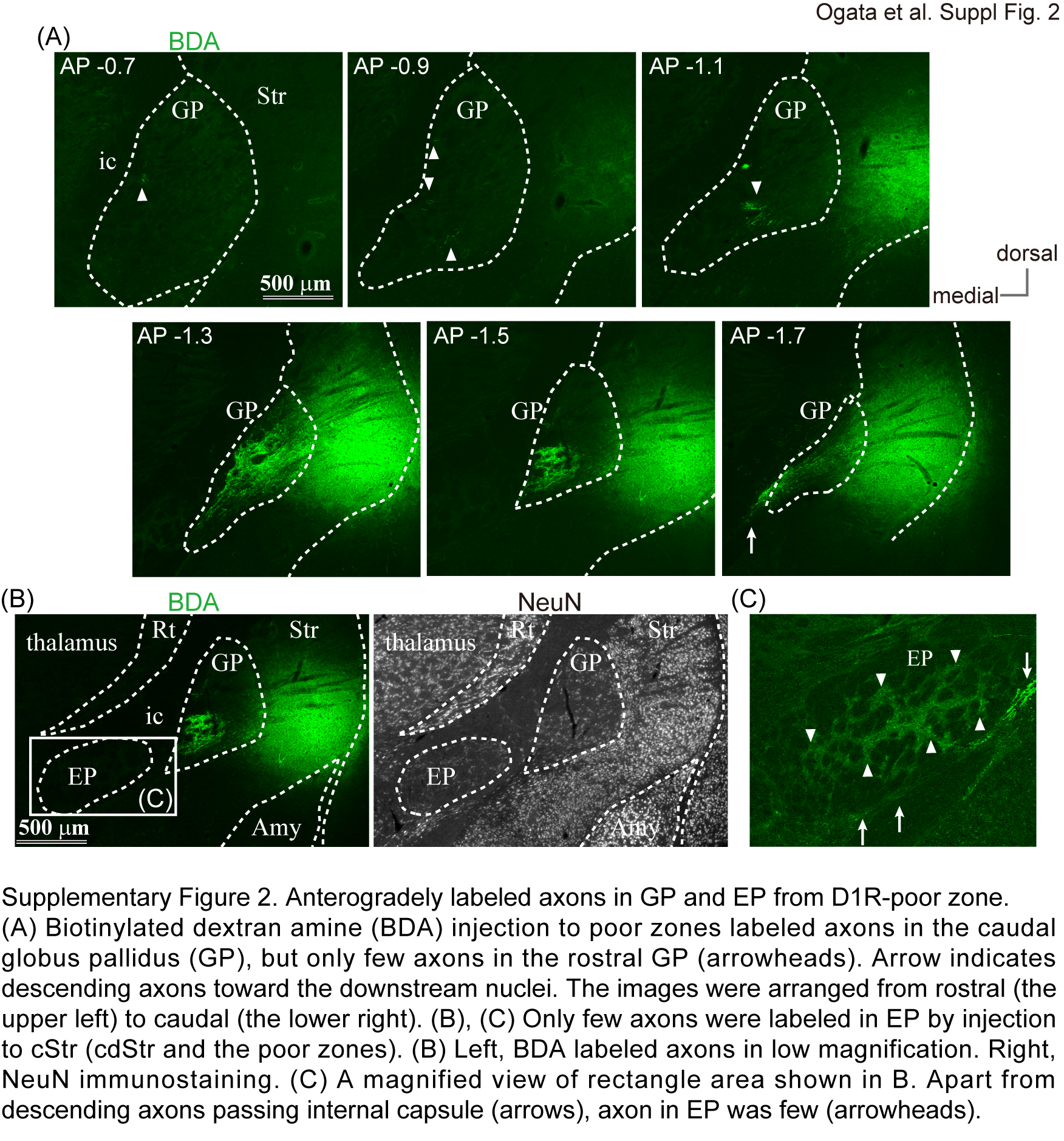

Supplement: Supplementary file 2 [file Image_2.tif]

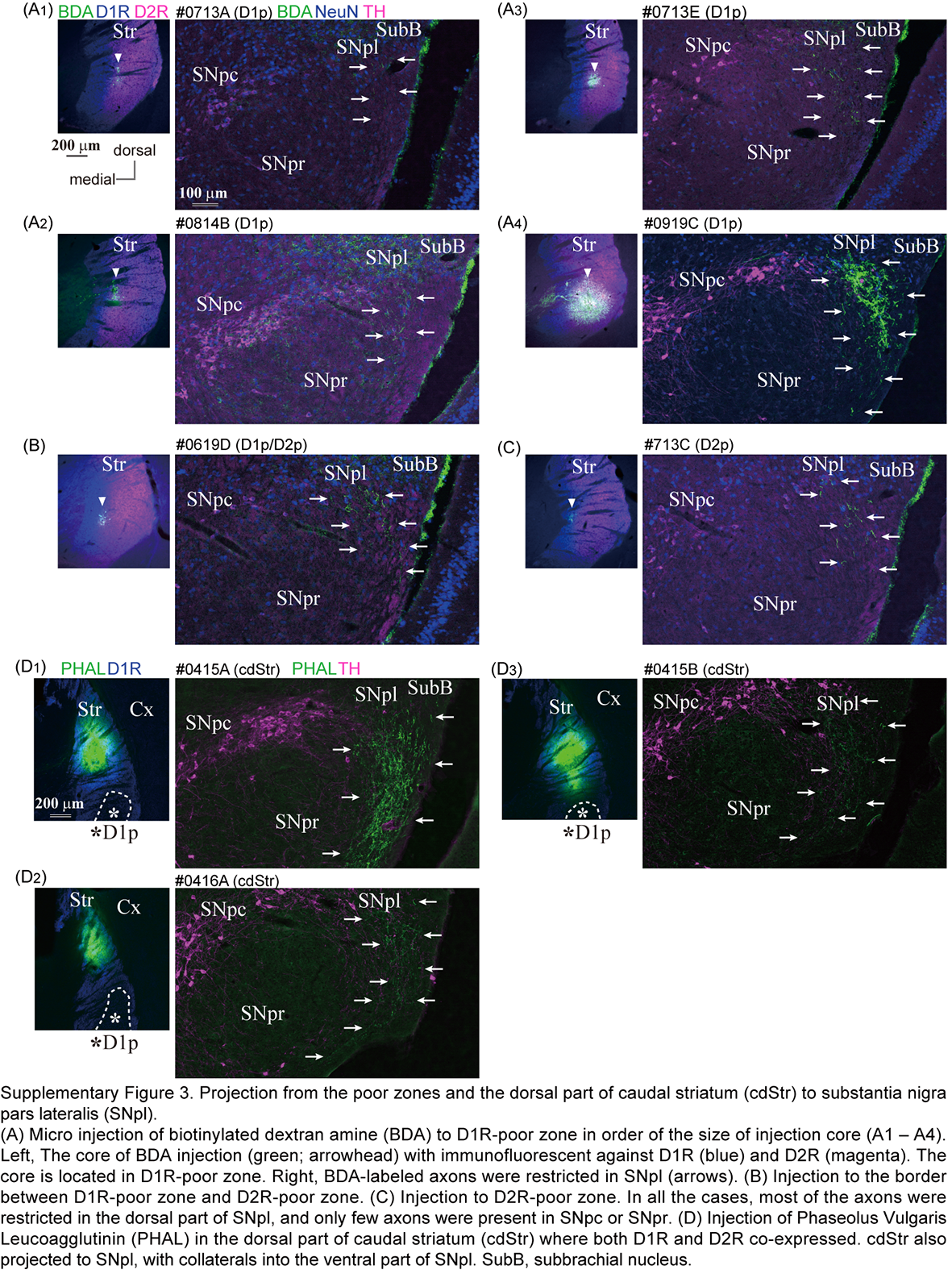

Supplement: Supplementary file 3 [file Image_3.tif]

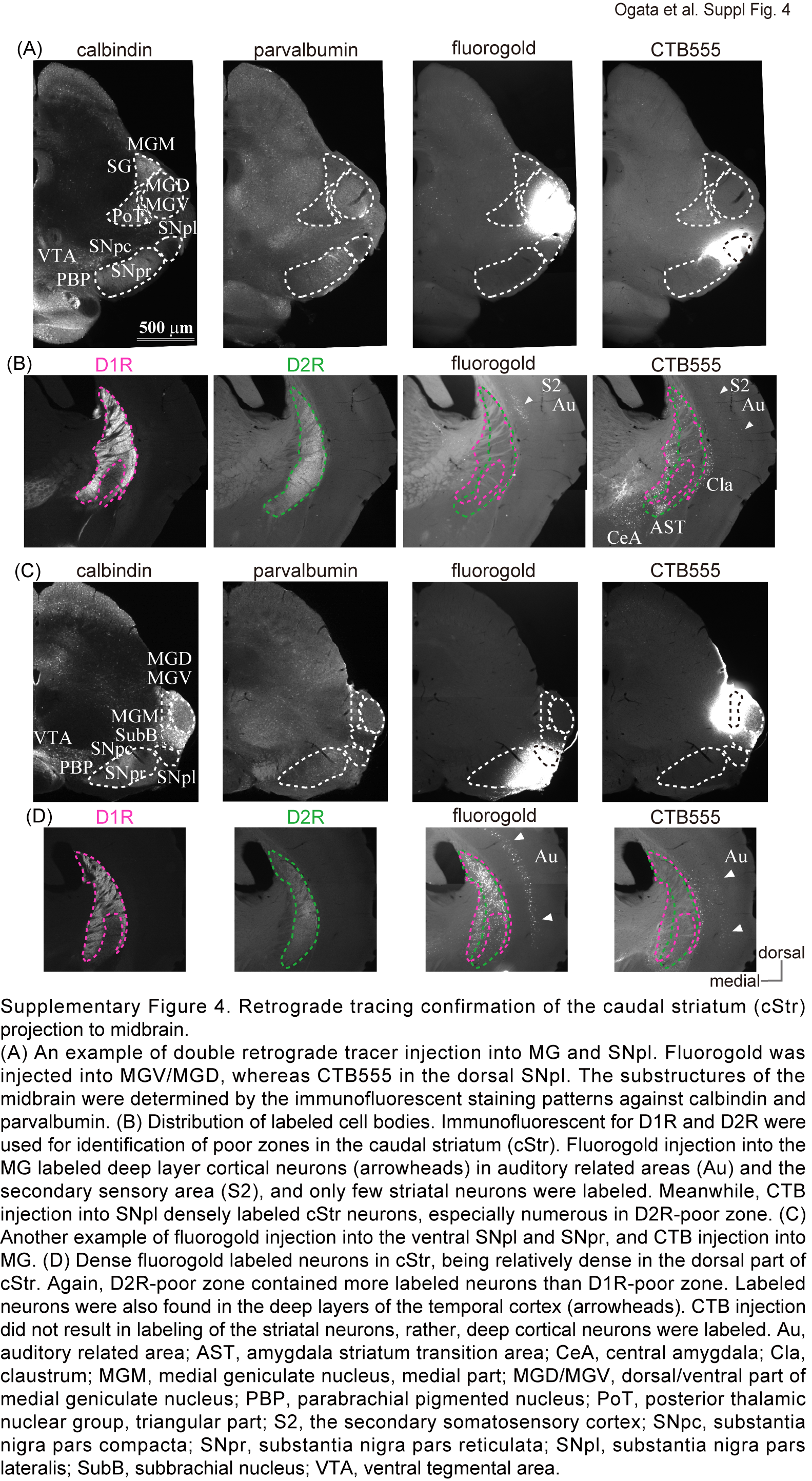

Supplement: Supplementary file 4 [file Image_4.tif]
